# Multidrug-resistance proteins are weak tumor associated antigens for colorectal carcinoma

**DOI:** 10.1186/1471-2172-12-38

**Published:** 2011-07-10

**Authors:** Christina S Mullins, Sven Eisold, Ernst Klar, Michael Linnebacher

**Affiliations:** 1 Department of General, Thoracic, Vascular and Transplantation Surgery, Section Molecular Oncology and Immunotherapy, University of Rostock, Schillingallee 35, 18055 Rostock, GERMANY

**Keywords:** Multidrug resistance, therapy resistance, tumor antigens, colorectal cancer, T cell epitopes, immunotherapy, reverse immunology

## Abstract

**Background:**

Multidrug resistance (MDR) is a clinically, highly relevant phenomenon. Under chemotherapy many tumors show an increasing resistance towards the applied substance(s) and to a certain extent also towards other agents. An important molecular cause of this phenomenon is an increased expression of transporter proteins. The functional relationship between high expression levels and chemotherapy resistance makes these MDR and MRP (MDR related protein) proteins to interesting therapeutic targets. We here wanted to systematically analyze, whether these proteins are tumor specific antigens which could be targeted immunologically.

**Results:**

Using the reverse immunology approach, 30 HLA-A2.1 restricted MDR and MRP derived peptides (MDP) were selected. Stimulated T cell lines grew well and mainly contained activated CD8^+ ^cells. Peptide specificity and HLA-A2.1 restriction were proven in IFN-γ-ELISpot analyses and in cytotoxicity tests against MDP loaded target cells for a total of twelve peptides derived from MDR-1, MDR-3, MRP-1, MRP-2, MRP-3 and MRP-5. Of note, two of these epitopes are shared between MDR-1 and MDR-3 as well as MRP-2 and MRP-3. However, comparably weak cytotoxic activities were additionally observed against HLA-A2.1^+ ^tumor cells even after upregulation of MDR protein expression by *in vitro *chemotherapy.

**Conclusions:**

Taken together, these data demonstrate that human T cells can be sensitised towards MDPs and hence, there is no absolute immunological tolerance. However, our data also hint towards rather low endogenous tumor cell processing and presentation of MDPs in the context of HLA-A2.1 molecules. Consequently, we conclude that MDR and MRP proteins must be considered as weak tumor specific antigens-at least for colorectal carcinoma. Their direct contribution to therapy-failure implies however, that it is worth to further pursue this approach.

## Background

Chemotherapy is, apart from resection and irradiation, the most common form of cancer treatment [1]. Unfortunately, many patients' tumors acquire drug resistance, including the classical multidrug resistance (MDR), during or after this kind of therapy. Therefore, this resistance against multiple even chemically and structurally unrelated chemotherapeutic agents, after treatment with a single drug, remains a major obstacle to overcome in the field of cancer therapy [1-3]. There are many different mechanisms of acquiring resistance including mutation or overexpression of the drug's targets as well as inactivation or efflux of the drug itself [4]. In the case of drug efflux, the MDR phenomenon is accompanied by the synthesis of P-glycoprotein, a member of the ATP-binding cassette (ABC) transporters. ABC transporters are channels or pumps using the energy of ATP hydrolyzation to drive the translocation of their substrates across membranes against diffusion gradients [3,4]. Hence, this mechanism allows cancer cells to survive cytotoxic or targeted therapies and treatment fails. In the process of acquiring resistance and onward, MDR proteins and MDR related proteins (MRP) are expressed in high levels on the cell surface.

Active immunotherapeutic approaches, such as dendritic cell [5,6] and peptide vaccinations [7,8] as well as passive immunotherapy, especially the application of therapeutic antibodies [9,10] have become indispensable as additional therapeutic options for various cancer entities in the last decade [11-13]. Active immunotherapeutic approaches mainly aim at the induction of cytotoxic T lymphocytes (CTL) which have the potential to eliminate even bulky tumor masses [14,15]. Yet, this ability raises concerns on the risk of deleterious autoimmune phenomena when breaking tolerance to self antigens [16]. In order to minimize this risk, the immunotherapeutic community is constantly looking for antigens which are tumor specific. The most desired features an ideal target for active immunotherapy would have to posses, are (I) virtually exclusive and (II) high density expression on the tumor cell, (III) involvement in the tumorigenic process or in tumor progression (to minimize the risk of immune escape variants), (IV) sufficient presentation in the form of MHC-bound peptides to allow for accessibility by CTL and finally (V) precursor T cells must be present in the T cell repertoire after negative selection [[17,18] with modifications].

Taking these features into consideration, MRP and MDR proteins completely fulfill the second and third criteria since they are highly expressed as a consequence of chemotherapy as described before. Moreover, Kuan *et al*. identified MRP-3 as a molecular target in glioblastoma [19] and developed a recombinant single-chain variable fragment antibody targeting MRP-3 [20]. Thus, the last two criteria posed to tumor antigens may also be met by MDR and MRP proteins.

Here, we took the classical reverse immunology approach of bioinformatic HLA-A2.1-restricted peptide prediction in combination with *in vitro *T cell stimulation in an autologous setting to address the questions: (I) can specific T cells be generated against several MDR and MRP proteins and additionally (II) can colorectal cancer (CRC) cells expressing MDR or MRP proteins be targeted by those T cells. We identified several HLA-A2.1-restricted T cell epitopes including two shared epitopes between two proteins and characterized the antitumoral potential of the generated CTL lines.

## Methods

### Cell culture

Human CRC cell lines (SW480, SW707, HCT-116 and Colo60H) and T2 cells (174 × CEM.T2 hybridoma, TAP1 and TAP2 deficient) were cultured in DMEM supplemented with 10% fetal calf serum, 2 mmol/L L-glutamine and 1% penicillin-streptomycin and incubated at 37°C and 5% CO_2_. All tumor cell lines were obtained from the tumor bank of the DKFZ (Heidelberg, Germany) or from the ATCC (Manassas, VA), media and supplements were purchased from PAA (Cölbe, Germany).

### CD40 ligand system for the culture of normal peripheral blood B-cells

Culturing of CD40 ligand-activated B cells (CD40 B cells) was performed as described [21]. Briefly, B cells from peripheral blood mononuclear cells (PBMC) were stimulated via NIH/3T3 cells stably expressing human CD154 (t-CD154). Lethally irradiated t-CD154 (100 Gy) were plated on 6-well plates (0.4 × 10^5 ^cells/well) and cultured overnight. After rinsing with PBS, PBMC (2 × 10^6 ^cells/ml) in Iscove's MEM (Gibco BRL) supplemented with 10% human AB serum, 5 μg/ml insulin, 50 μg/ml transferring and 15 μg/ml gentamicin were added and cultured in the presence of IL-4 (2 ng/ml; Cellgenix, Freiburg, Germany) and cyclosporin A (5.5 × 10^-7 ^M). At intervals of 3 to 5 days, cells were transferred to new plates containing fresh irradiated t-CD154 cells.

### Peptides and HLA-A2.1-binding assay

The specific computer program SYFPEITHI [22] (access via: http://www.syfpeithi.de) was applied to predict peptides displaying HLA-A2.1-binding motifs from the protein sequences of MDR and MRP proteins (MDPs; see Table 1 for details). Peptides were purchased from the peptide synthesis unit of the DKFZ. Stock solutions (5 mg/ml in DMSO) were stored at -70°C and diluted to 500 μg/ml in PBS before use. T2 cells were pulsed with 10 μg/ml peptide and 5 μg/ml β2-microglobulin (Sigma, Deisenhofen, Germany) overnight at 37°C. HLA-A2.1 expression was analysed by flow cytometry using MAb BB7.2 followed by incubation with a FITC-conjugated goat Ab binding anti-mouse Ig (Dako, Hamburg, Germany).

**Table 1:** Details of the MDR and MRP-derived peptides and the MDP-specific T cells

Protein	**Accession Number**^**1**^	Name	**Peptide**^**2**^	**SYFPEITHI Score**^**3**^	**Fluorescence Index**^**4**^	INFγ **ELISpot**^**5**^	Cytotoxicity **Test**^**6**^
Influenza Matrix Protein	AAA43682	MP	^57^-GILGFVFTL	30	0,80		
Growth Regulated Protein P68	226021	P68	^128^-YLLPAIVHI	30	0,93		
MDR-1	AF016535	MDP01	^686^-ALDESIPPV	29	2,07	**0,61**	
		MDP02	^218^-ILAISPVLGL	30	0,17	0,27	
		MDP03	^858^-LLLLAIVPII	27	0,96	0,17	
		MDP04	^551^-LLLDEATSAL	26	0,64	0,30	
		MDP05	^850^-FIYGWQLTLL	26	0,22	**9,94**	89,7
MDR-3	NM_018849	MDP04	^551^-LLLDEATSAL	26	0,64	0,30	
		MDP05	^850^-FIYGWQLTLL	26	0,22	9,94	89,7
		MDP06	^467^-YLREIIGVV	28	0,13	**0,60**	
		MDP07	^833^-ALIAQNIANL	30	0,25	**0,99**	44,2
		MDP08	^860^-LLAVVPIIAV	29	0,64	**1,75**	62,9
MRP-1	L05628	MDP09	^1224^-SLSAGLVGL	31	0,04	0,37	
		MDP10	^452^-ILALYLLWL	30	0,17	0,34	
		MDP11	^745^-ALLPDLEIL	30	0,61	**0,52**	88,2
		MDP12	^1109^-LLATPIAAI	30	0,39	**0,80**	
		MDP13	^118^-LLATFLIQL	29	0,42	0,02	
		MDP14	^508^-ILNGIKVLKL	32	0,27	0,08	
		MDP15	^461^-NLGPSVLAGV	29	0,49	0,42	65,8
		MDP16	^466^-VLAGVAVMVL	29	0,24	0,06	
MRP-2	NM_000392	MDP17	^661^-IMAGQLVAV	31	0,42	0,04	
		MDP18	^783^-LLDDPLSAV	29	1,55	**1,57**	80,2
		MDP19	^42^-LLAPWQLLHV	31	0,21	**1,41**	29,4
		MDP20	^77^-ILAAIELALV	30	0,50	**0,55**	
		MDP21	^782^-YLLDDPLSAV	30	1,49	**0,77**	
MRP-3	AF104943	MDP14	^508^-ILNGIKVLKL	32	0,27	0,08	
		MDP15	^461^-NLGPSVLAGV	29	0,49	0,42	65,8
		MDP18	^783^-LLDDPLSAV	29	1,55	1,57	80,2
		MDP22	^1220^-SLNPGLVGL	34	0,64	**1,07**	
		MDP23	^1020^-ILQGFLVML	31	1,33	0,47	
		MDP24	^1082^-VLAPVILML	30	1,98	0,50	
		MDP25	^422^-DLAPFLNLL	29	0,50	0,06	
		MDP26	^1115^-ILPLAVLYTL	29	0,23	0,18	
MRP-5	XM_002914	MDP27	^304^-AILGMIYNV	29	1,09	0,37	
		MDP28	^784^-LLLGETPPV	29	1,35	**0,67**	24,9
		MDP29	^296^-LLAGGPVVAI	31	0,23	**5,07**	97,04
		MDP30	^1237^-GMALFRLVEL	29	0,26	0,05	

^1 ^Protein or nucleotide accession numbers are indicated

^2 ^Position of the start amino acid in the protein is indicated

^3 ^Predicted binding scores to HLA-A2.1 using computer assisted analysis

^4 ^(Mean fluorescence with peptide-mean fluorescence without peptide)/(mean fluorescence without peptide)

^5 ^Percentage of cells in the MDP-specific T cell bulk cultures secreting IFN-γ upon stimulation with peptide-loaded T2 cells (maximum values observed over time of stimulation)

^6 ^Lysis of MDP-loaded T2 cells in % at an effector to target cell ratio of 100:1

Results are representative of at least two experiments.

MDPs derived from more than one protein are listed twice with lighter shading when listed the second time.

### T-cell purification and induction of peptide-specific cytotoxic T lymphocytes (CTL)

Whole CD3^+ ^T cells were isolated from PBMC by magnetic depletion of non T cells using the MACS Pan T Cell Isolation Kit II (Miltenyi-Biotec, Bergisch Gladbach, Germany) according to the manufacturer's instructions. Preparations contained at least 97% of CD3^+ ^cells as assessed by immunophenotypic analysis. CD40 B cells of healthy HLA-A2.1^+ ^donors were incubated with 10 μg/ml of different MDP-mixes (Table 2) in serum-free Iscove's MEM for 1 hr at room temperature, washed twice to remove excess peptide, irradiated (30 Gy) and added to purified CD3^+ ^autologous T cells at a ratio of 1:4 (T-cells:CD40 B cells) in Iscove's MEM containing 10% human AB serum, supplements (1:100) and hIL-7 (10 ng/ml, Cellgenix). T cells were plated in 24-Well plates at a density of 2 × 10^6 ^T cells/well in 1 ml medium. After 3 days of culture 1 ml complete medium was added. For T cell restimulation the procedure was repeated on a weekly basis. IL-2 was added at days 21 (10 IU/ml, Proleukine^®^) and 24, and from day 28 on only hIL-2 was used.

**Table 2:** Composition of the MDP mixes used for bulk T cell stimulation

Peptide Mix	MDPs	**Proteins**^**1**^
Mix-1	MDP01-MDP03 MDP04, MDP05	MDR-1 MDR-1, MDR-3
Mix-2	MDP06-MDP08 MDP27, MDP28	MDR-3 MRP-5
Mix-3	MDP09-MDP13	MRP-1
Mix-4	MDP14, MDP15 MDP16 MDP29, MDP30	MRP-1, MRP-3 MRP-1 MRP-5
Mix-5	MDP17, MDP19-MDP21 MDP18	MRP-2 MRP-2, MRP-3
Mix-6	MDP22-MDP26	MRP-3

^1 ^MDR or MRP protein the listed MDP originates from MDP representing epitopes shared between two MDR or MRP proteins are underlined.

### Enzyme-linked immunospot (ELISpot) assay

ELISpot assays were performed as described before [23]. Briefly, nitrocellulose 96-well plates (Multiscreen; Millipore, Bedford, MA) were covered with mouse anti-human IFN-γ MAb (Mabtech, Nacka Strand, Sweden) and blocked with medium containing serum. Varying numbers of effector cells were plated in triplicates with 3.5 × 10^4 ^peptide-loaded T2 cells per well as targets. After incubation for 16 h, plates were washed, incubated with biotinylated rabbit anti-human INF-γ secondary antibody, washed again, incubated with streptavidin-coupled alkaline phosphatase, followed by a final washing step. INF-γ-secreting cells were visualized by incubation with NBT/BCIP (Sigma) for 45 min, reaction was stopped with water and spots were counted.

### Cytotoxicity assay

Cytotoxicity assays were performed as described before [21]. Briefly, effector T cells were incubated in triplicate in 96-well plates with ^51^Cr-labeled target cells at a ratio of 3-100:1 (E:T). Cells were incubated for 4 h (T2 cells) and 8 h (colorectal cancer cell lines) at 37°C. Plates were centrifuged, and aliquots of the supernatants were harvested and counted in a γ-counter. Percent cytotoxicity was calculated as follows: 100% × (experimental release-spontaneous release)/(maximal release-spontaneous release).

### Induction of resistance towards chemotherapeutic agents

CRC cell lines were cultured as described above. For augmentation of MDR and MRP protein expression on the cell surface, cells were treated with 5-FU (500 ng/ml-50 μg/ml), cisplatin (50 ng/ml-5 μg/ml) and a combination of both. MDR and MRP expression levels were assessed with qPCR using the LightCycler^® ^technology (Roche, Mannheim, Germany) as described [24,25].

## Results

### MDR and MRP-derived peptides

A total of thirty peptides displaying HLA-A2.1-binding motifs were selected from the protein sequences of MDR-1 and MDR-3, MRP-1, MRP-2, MRP-3 and MRP-5 (MDPs; see Table 1 for details). Their binding capacity to HLA-A2.1 was first tested in a functional binding assay using T2 cells expressing HLA-A2.1 as the sole MHC-molecule on their surface. Additionally, these HLA-A2.1 molecules are devoid of bound endogenous peptide due to a processing defect of the T2 cells. Empty HLA molecules exhibit a rapid turnover and thus T2 cells express only low levels of HLA-A2.1. Exogenously added peptides can stabilize the HLA-A2.1 molecules and thus augment the HLA-A2.1 expression levels assessed by flow cytometry. This is a good functional measure for HLA-A2.1 binding capacity of peptides [26]. As expected, the thirty MDPs displayed varying HLA-A2.1-binding capacity. Details can be depicted from Table 1.

### Stimulation with MDP-mixes leads to CTL proliferation

**Figure 1:**
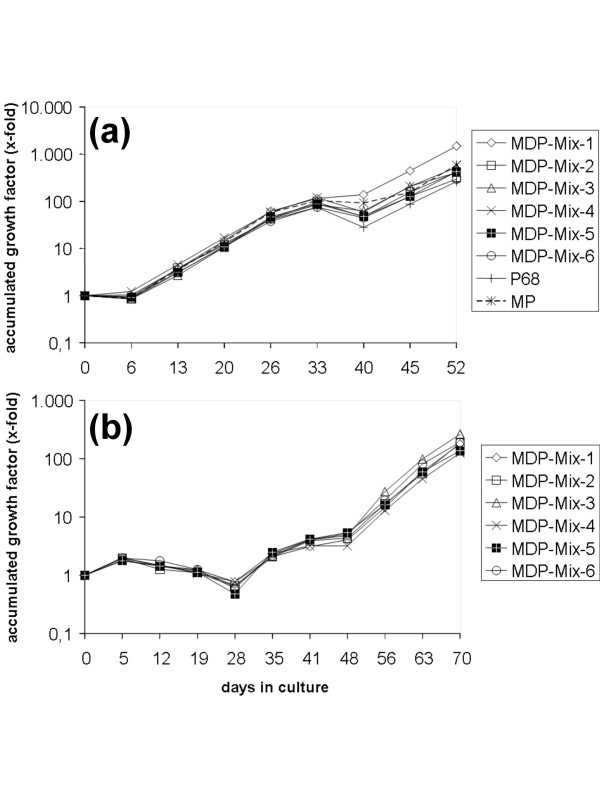
**Growth of MDP-Peptide-Mix stimulated T cells**. The outgrowth of MDP-stimulated T cells was assessed by counting the number of viable T cells weekly and calculating an accumulated growth factor. T cell cultures were generated from two healthy donors; (A) donor 1 and (B) donor 2.

Next feasibility of induction and antigen recognition of MDP-specific CTL should be determined. Therefore, PBMC of two healthy donors were used to isolate T cells and to generate CD40 B cells. The latter were loaded with MDPs in different mixes and used for stimulation of autologous T cells. T cell proliferation rates were determined over time (donor 1: Figure 1A; donor 2: Figure 1B). Stimulations using the influenza matrix peptide MP (positive control) and the P68 peptide (negative control) were additionally performed for donor 1 and served as controls (Figure 1A). All of these conditions clearly resulted in sustained T cell proliferation, with MDP mix 1 (donor 1: Figure 1A) and MDP mix 3 (donor 2: Figure 1B) inducing the highest proliferation. Outgrowing T cell cultures were predominantly CD8^+ ^(data not shown).

### IFN-γ producing CTL are induced by MDP stimulation

**Figure 2:**
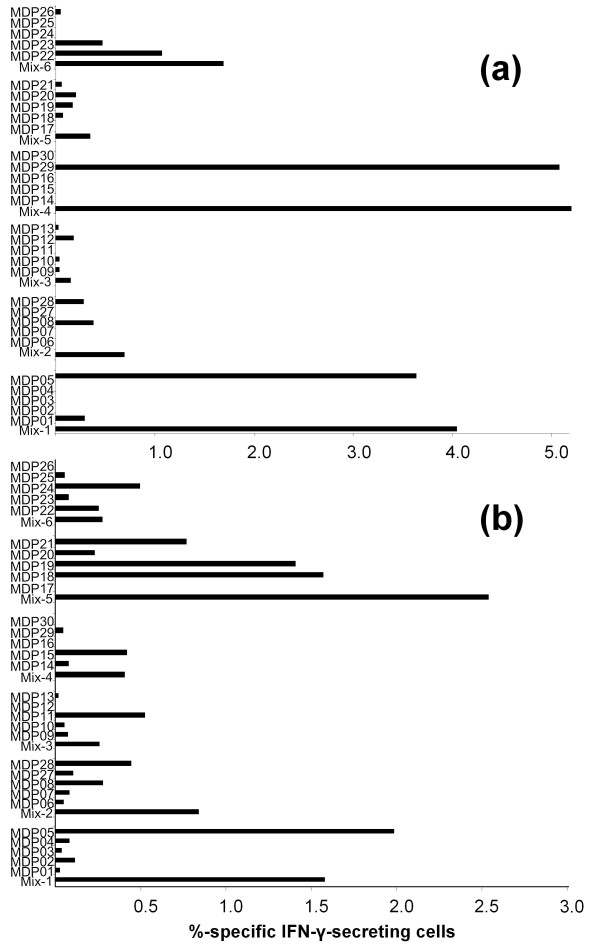
**ELISpot analysis of MDP-specific IFN-γ release**. The percentage of T cells secreting IFN-γ in response to MDP-loaded target cells was determined in a series of ELISpot experiments. Reactivity for MDP-stimulated T cells of (A) donor 1 (exemplarily for day 77) and (B) donor 2 (exemplarily for day 77) is given. Analysis was performed in triplicates with 1.000 effector and 10.000 target cells per well.

The rate of peptide specific stimulation was investigated by IFN-γ-ELISpot analyses. T2 cells were loaded with the respective peptides used for T cell stimulation (either single peptides or as mixes) and served as targets for the IFN-γ-ELISpot analyses. Significant numbers of IFN-γ secreting T cells could be detected and therefore hint towards a strong reactivity against several peptides for both donors (Figure 2 and Table 1). In summary, peptide mixes 1, 2, 4, 5 and 6 led to IFN-γ producing CTL with a total of 14 MDPs being recognized by more than 0.5% of the T cells in the respective cultures. The greatest effects were seen with MDP mixes 1 and 4 for donor 1 and peptide mixes 1 and 5 for donor 2. When looking at the single peptide levels MDP05 (mix 1), MDP08 (mix 2) and MDP29 (mix 4) for donor 1 and MDP05 (mix 1) and MDP18 and 19 (mix 5) for donor 2 provoked the strongest reactions (Figure 2 and Table 1).

### MDP-specific T cells have strong cytolytic potential

**Figure 3:**
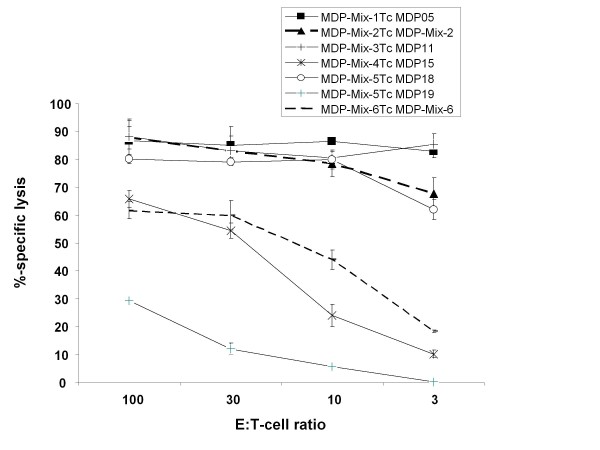
**Analysis of MDP-specific CTL activity against peptide-sensitized T2 target cells**. Cytotoxic activity of MDP-specific CTL after at least 3 rounds of restimulation with the indicated MDP mixes was analyzed in standard ^51^Cr-release assays. MDP-specific reactivity of T cell bulk cultures (Mix-1Tc to Mix-6Tc) was tested against T2 target cells loaded with either the indicated peptide mixes or the given individual MDP. Effector T cells were added at different effector to target cell (E:T) ratios. A representative experiment with CTL derived from donor 2 is shown. The analysis was performed in triplicates with 1.000 effector cells per well and after a 6 h incubation period.

IFN-γ production is a clear sign of specific activation. It does, however, not prove cytotoxic ability. Therefore, we next tested the potential of the induced CTL to kill peptide-loaded T2 target cells. Here, we observed efficient target cell killing when peptides used for T cell stimulation and target cell loading matched (Figure 3 and Table 1). We observed no reactivity above ten percent background when irrelevant peptide was loaded onto the T2 targets (data not shown). Overall, the results of ELISpot and cytotoxicity tests showed a high degree of accordance. However, two details are remarkable: (i) despite relatively few IFN-γ producing CTL upon cognate stimulation with MDP11 (donor 2; 0.52%), there was a comparably high reactivity observed in the cytotoxicity test and (ii) in contrast, approximately 1.4% donor 2 mix-5 stimulated T cells secreted IFN-γ upon stimulation with MDP19 but they did only marginally lyse MDP19-loaded T2 targets (Figure 2B and Figure 3).

### MDP-specific CTL lyse tumor cells

**Figure 4:**
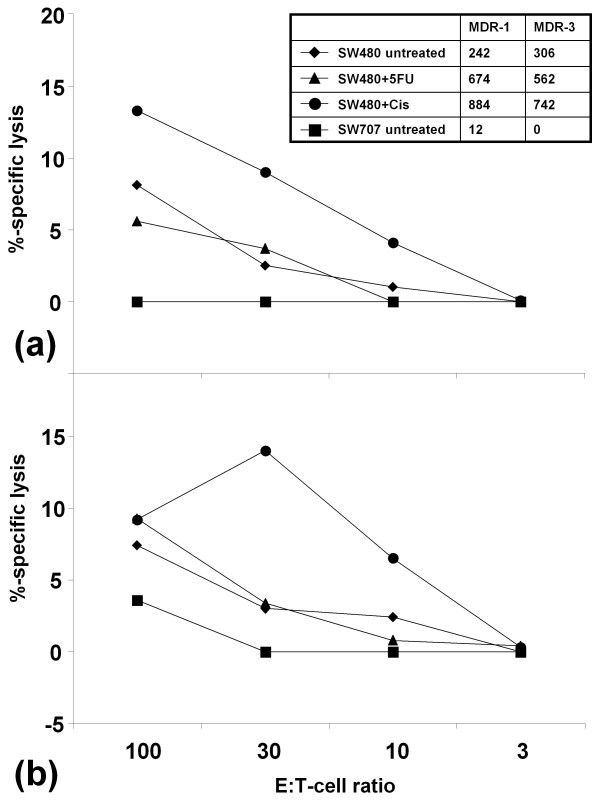
**Analysis of MDP-specific CTL activity against CRC cell lines**. Standard ^51^Cr-release cytotoxicity assays were performed with MDP05-specific CTL using CRC cell lines as target cells. Effector T cells were added at different effector to target cell (E:T) ratios. A representative experiment of CTL derived from (A) donor 1 and (B) donor 2 is shown. Analysis was performed in triplicates with 1.000 effector cells per well and after a 6 h incubation period. The expression levels of MDR-1 and MDR-3 are given as additional information (right upper corner) for untreated SW480 and SW707 and for cisplatin and 5-FU pretreated SW480 in copies/μl cDNA normalized to the housekeeping genes cyclophilin B and hypoxanthine guanine phosphoribosyltransferase.

The ability to kill tumor cells endogenously expressing the target peptides is the final goal of the reverse immunology approach. We thus analyzed in further cytotoxicity tests, whether MDP-specific CTL really attack tumor cells. The target tumor cells were pretreated with increasing doses of cisplatin and 5-FU *in vitro *(data not shown). Then, cultures showing high level expression of MDR and MRP were chosen as target cells. Here, tumor cell lysis rates of up to 8% could be achieved for the HLA-A2.1^+ ^cell line SW480 and CTL specific for MDP-mix 1 (Figure 4 and 4B). Respectively lysis rates of up to 17% for MDP-mix 3 and 5% for MDP-mix 5 could be obtained (data not shown). However, these were the strongest reactions observed; even lower levels of tumor cell killing were found for other HLA-A2.1^+ ^target cell lines (HCT116, SW707 and Colo60H).

In summary, the observed cytotoxic effects were rather low.

## Discussion

Physiologically, MDR and MRP proteins are expressed in a variety of human tissues including liver, kidney and the blood-brain-barrier [[27] and [28]]. Considering their function as membrane transporters with the ability to transport different substances against concentration gradients their function obviously lies-at least to a greater proportion-in cellular detoxification [[27] and [28]]. Of note, it has been suggested that MDR and MRP proteins are expressed particularly in tissue stem cells and thus protect those precious cells from damage [4].

When looking at tumor, the expression of MDR and MRP proteins has been reported for malignant cells of different entities [29]. Clinical data prove that their expression is upregulated as an attempt to acquire resistance towards the effects of chemotherapeutic agents in the cause of chemotherapy [30]. To us, this phenomenon is in perfect accordance with the observation that tumor cells contain properties of the tissue stem cell they originate from, mainly unlimited life span, resistance towards apoptosis and cellular plasticity [31].

This detailed mechanistic understanding of the MDR and MRP protein function in malignant cells suggests those proteins as perfect targets for the development of novel therapeutical approaches. Beside classical drugs, small molecules and therapeutical antibodies, immunotherapeutic strategies steadily gain more clinical relevance. A magnitude of immunological target proteins relevant for tumorigenesis and maintenance of the transformed state have been identified so far. Those include Her2/neu, bcr/abl, WT-1, survivin and hTERT [8,32-35]. Nakai *et al*. correlated enhanced MDR-1 expression with chemoresistance of cancer stem cells derived from glioblastoma and suggested MDR-1 as an immunotherapeutic target [29]. Kuan and coworkers suggested MRP-3 as a potential immunotherapeutic target for glioblastoma [19]. They subsequently even developed a specific therapeutic antibody [20]. Clinical testings have, however, not yet been reported. Similarly, Yamada *et al*. identified MRP-3 as a true tumor rejection antigen when analyzing the target structure of a cytotoxic T cell clone isolated from a human lung cancer patient [36].

Taking these considerations as a starting point, we chose the classical *in silico *prediction to identify MDR and MRP-derived T cell epitopes restricted to HLA-A2.1 since this is the most frequent HLA allele in the Caucasian population. A comprehensive number of epitope peptides was selected and subsequently tested for immunostimulatory potential. This has, to the best of our knowledge, not been investigated before. Testing was performed in mixed-lymphocyte-peptide cultures with T cells from two healthy individuals. Satisfactory growth of T cell cultures *in vitro *was observed. Phenotypical analysis revealed the outgrowth of predominantly CD8^+ ^CTL. Subsequent functional testing could identify reactivity against twelve peptides out of thirty tested MDPs. MDR and MRP-specific CTL recognized their epitopes in IFN-γ ELISpots and in cytotoxicity tests using peptide-loaded HLA-A2.1^+ ^target cells. Of note, we obtained CTL specific for epitopes derived from all MDR and MRP proteins included into the analysis, namely MDR-1, MDR-3, MRP-1, MRP-2, MRP-3 and MRP-5. Strikingly, two of the epitopes are shared between MDR-1 and MDR-3 (MDP05) as well as MRP-2 and MRP-3 (MDP18), which may be of special interest for future immunological analysis.

Collectively, this allows the conclusion that the human immune system harbors HLA-A2.1-restricted T cells specifically recognizing MDR and MRP-derived peptides. Additionally, it is in line with the previous description of HLA-A24-restricted T cells specific for MRP-3 [36]. The combination of these studies leads to the conclusion that no absolute central and peripheral tolerance seems to exist for MDR and MRP proteins.

However, when testing the CTL's potential to kill tumor cell lines expressing high levels of MDR and MRP proteins, the level of tumor cell recognition was disappointingly low in the present study. This may best be explained by insufficient or missing processing of MDR and MRP-proteins by the cellular proteasomal machinery or similarly by insufficient or missing presentation in the context of HLA-A2.1 molecules. Our screen was limited to HLA-A2.1, and consequently MDR and MRP-derived peptides may be presented in high levels in other HLA-molecules. Moreover, our findings are to some extent in contrast to the observations of Yamada *et al*. They could activate T cells with MRP-3 derived peptides in an HLA-A24 restricted manner and further observed a specific lysis of lung cancer cells with a maximum of 30% lysis at an E:T ratio of 80:1 [36]. We, however, observed a lower level of tumor cell killing (approximately 15% at an E:T ratio of 100:1) and since our screen was limited to CRC cell lines, this may hint towards comparably bad processing and presentation of MDR and MRP proteins in CRC cells. Intrinsic resistance towards CTL lysis of the CRC cell lines used in our study can be excluded, since we successfully used them as CTL targets before [21,23]. A speculative hypothesis is that this may be the result of previous immunoediting of the colorectal cancer cells. This would imply a high relevance of MDR and MRP as tumor specific antigens in the natural course of host and tumor immune interaction but would clearly limit the usefulness of HLA-A2.1-restricted peptides for clinical immunotherapy.

## Conclusion

MDR and MRP-derived peptides can give rise to completely functional CTL out of the human repertoire. Killing of CRC tumor cells was however only marginally. This does not recommend the targeting of MDR and MRP proteins as solitary tumor antigens in immunotherapeutic interventions BUT because of their therapy-related up regulated expression they may well be considered as add-on in multi-epitope immunotherapies. This must be assessed in future studies. Yet, they may be perfect tumor antigens for other tumor entities.

## List of abbreviations

ABC: ATP-binding cassette; CD40 B cells: CD40 ligand-activated B cells; CRC: colorectal cancer; CTL: cytotoxic T lymphocytes; MDR: multidrug resistance; MDP: MDR and MRP derived peptides; MRP: MDR related proteins; t-CD154: NIH/3T3 cells stably expressing human CD154; PBMC: peripheral blood mononuclear cells.

## Competing interests

The authors declare that they have no competing interests.

## Authors' contributions

CSM helped to analyse and interpret the data and drafted the manuscript. SE performed part of the analysis and together with EK helped to draft the manuscript and reviewed the manuscript. ML conceived the study, and participated in data analysis and in writing of the manuscript. All authors red and approved the final manuscript.
